# Impact of Long-Term Enzyme Replacement Therapy on Glucosylsphingosine (Lyso-Gb1) Values in Patients with Type 1 Gaucher Disease: Statistical Models for Comparing Three Enzymatic Formulations

**DOI:** 10.3390/ijms22147699

**Published:** 2021-07-19

**Authors:** Tama Dinur, Ulrike Grittner, Shoshana Revel-Vilk, Michal Becker-Cohen, Majdolen Istaiti, Claudia Cozma, Arndt Rolfs, Ari Zimran

**Affiliations:** 1 Shaare Zedek Medical Center, Gaucher Unit, Jerusalem 9103102, Israel; dinurtama@gmail.com (T.D.); michalbc@szmc.org.il (M.B.-C.); joleenist@szmc.org.il (M.I.); azimran@gmail.com (A.Z.); 2 Berlin Institute of Health, Charité—Universitätsmedizin Berlin, 10117 Berlin, Germany; urlike.grittner@charite.de; 3 Institute of Biometry and Clinical Epidemiology, Charité—Universitätsmedizin Berlin, 10117 Berlin, Germany; 4 Centogene AG, 18055 Rostock, Germany; Claudia.cozma@centogene.com; 5 Faculty of Medicine, Hebrew University of Jerusalem, Jerusalem 9112002, Israel; 6 Medical Faculty, University of Rostock, 18051 Rostock, Germany; arndt.rolfs@web.de

**Keywords:** glucosylsphingosine, lyso-Gb1, Gaucher disease, biomarkers, enzyme replacement therapy

## Abstract

For three decades, enzyme replacement therapy (ERT), and more recently, substrate reduction therapy, have been the standard-of-care for type I Gaucher disease (GD1). Since 2012, three different ERTs have been available. No clinical trial or academic study has ever compared these ERTs beyond one year. Herein we compare the impact of the ERTs on repeated measurements of glucosylsphingosine (lyso-Gb1; the most sensitive and GD-specific biomarker). A total of 135 adult patients (77 (57%) female) with GD1, followed from July 2014 to March 2020 and treated with a single ERT (imiglucerase (n = 41, 30.4%), taliglucerase alfa (n = 21, 15.6%) and velaglucerase alfa (n = 73, 54.1%)), were included. Disease severity was defined by genotypes (mild: N370S (c.1226A>G) homozygous and N370S/R496H (c.1604G) compound heterozygous; severe: all other genotypes) and by the severity score index (SSI; mild: <7; severe: ≥7). Lyso-Gb1 testing was performed at Centogene™ on dry blood spot samples collected during routine visits. Patients treated with imiglucerase had higher lyso-Gb1 levels at different time points. A huge variation in lyso-Gb1 levels was noticeable both inter-individually and intra-individually for all three ERTs. A steeper and faster decrease of lyso-Gb1 levels was shown in velaglucerase alfa. Nevertheless, the differences between medications were not very large, and bigger numbers and more pretreatment data are required for more powerful conclusions.

## 1. Introduction

Gaucher disease (GD) is an autosomal recessive lysosomal storage disorder caused by partial deficiency of the lysosomal enzyme β-glucocerebrosidase, leading to accumulation of glucocerebroside in tissue macrophages, thereby mainly affecting the spleen, liver, and bone marrow [1]. The disease is pan-ethnic and has an estimated prevalence of 1:50,000–100,000 in the general population, but with a predilection for Ashkenazi Jews, wherein 1:17 are carriers and about 1:800 is affected [2].

Gaucher disease has been traditionally divided into three clinical forms: type 1 (GD1), a chronic, non-neuronopathic form, defined by the absence of central nervous system (CNS) involvement (adult type); type 2 (GD2), an acute and invariably fatal neuronopathic form in infants/toddlers (infantile type); and type 3 (GD3), a sub-acute neuronopathic form (juvenile form) [1]. The great phenotypic heterogeneity of GD is explained in part by the many (>860) mutations identified to date within the glucocerebrosidase gene (*GBA1*), but also by different genetic, epigenetic, and environmental factors [3]. Additionally, many comorbidities impact the clinical features of GD, some of which are significantly more common in GD than in the general population, such as Parkinson’s disease and various hematological disorders [4].

In GD1, despite the genotype-phenotype correlations that have been reported and some relationship between a biomarker value and clinical severity that has been noted [5,6], we are still unable to predict the clinical course of an individual patient at the time of diagnosis, whether prenatally or at a very young age. For the neuronopathic forms, there is also a significant diversity that may impact medical decisions related to the continuation of pregnancy or selection of therapy, particularly investigational ones, as no specific treatment is currently available for types 2 and 3 [7].

At present, there are several formulations of enzyme replacement therapy (ERT), all administered intravenously, usually once every two weeks, and two compounds of oral substrate reduction therapy (SRT) [8]. The magnitude of response (reduction in spleen and liver volume, improvement in blood counts and other laboratory parameters, and especially the changes in the bony features) cannot be accurately predicted regardless of age, gender, genotype, and baseline findings [9]. It is to be hoped that a suitable biomarker will be identified that would be able to provide information on treatment response that may have ramifications to indications of therapy, timing to begin, choice of modality, and dosage.

The biomarkers that have been used until recently included the older and less specific ones such as angiotensin-converting enzyme (ACE), tartrate-resistant acid phosphatase (TRAP), serum ferritin, and high-density lipoprotein (HDL), and the newer, more specific, but still imperfect, ones, the chitotriosidase and CCL18. In a search for a more specific and sensitive biomarker, two groups independently identified glucosylsphingosine (lyso-Gb1) as a candidate biomarker, which has been confirmed to be 100% specific and 100% sensitive [10,11]. 

The current study was designed to evaluate the impact of the three ERTs commercially available in Israel on lyso-Gb1 levels in patients with GD1 from a single center in Jerusalem according to disease severity and to examine possible differences between the formulations.

## 2. Results

### 2.1. Study Cohort

In this longitudinal observational study, 135 patients (77, 57% female) with GD1 were on ERT at the first observation time point over a median period of 51 months (range: 0–322 months) (Table 1).

Most patients (65%) had a milder version of GD1 according to the genotype (c.1226A>G homozygous or c.1604G heterozygous) and SSI (mean: 6, SD: 4). All patients were on the same ERT during the whole study period: most on velaglucerase alfa (73, 54.1%), 41 (19.2%) on imiglucerase, and 21 (15.6%) on taliglucerase alfa (Table 1). Patients on imiglucerase had a more severe phenotype (based on SSI) and genotype and were on ERT for a longer time at the first study time point compared to patients on velaglucerase alfa and taliglucerase alfa (Table 1). 

### 2.2. First Lyso-Gb1 Observation

Associations of lyso-Gb1 with patient characteristics at the first time point are presented in Table 2. Patients older than 65 years had lower lyso-Gb1 levels compared to patients younger than 35 years. The lowest lyso-Gb1 levels were found in samples taken after 56 to 80 months of ERT compared to shorter and longer ERT duration (Table 2). However, since there is a lot of intra-individual variability of measures and high variation of observation periods between individuals, it is unclear if there is indeed an increase in lyso-Gb1 after a longer treatment period.

### 2.3. Longitudinal Lyso-Gb1 Observations

**Figure 1:**
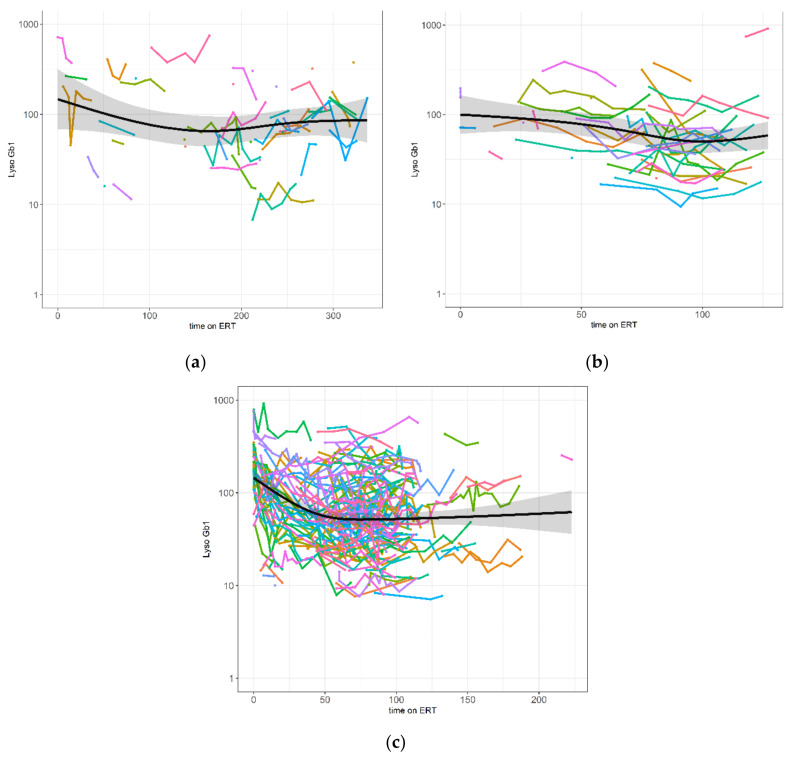
Longitudinal lyso-Gb1 values over time (months) on the three enzyme replacement therapies (ERTs); (**a**) Imiglucerase (41 individuals, 126 measures), (**b**) Taliglucerase alfa (21 individuals, 90 measures), and (**c**) Velaglucerase alfa (73 individuals, 407 measures). Colored lines are aligned to the individual time on ERT at the time of lyso-Gb1 measurement. The black line and grey area indicate the unadjusted regression line (with 95% confidence interval (CI)).

Figure 1 shows the original data of changes of lyso-Gb1 on the three ERTs aligned to the time on ERT. For all three ERTs, patients had lower lyso-Gb1 values after being some time on therapy compared to the starting phase reaching a plateau around 100 months (~8 years) on treatment. However, a huge variation in lyso-Gb1 values was noticeable both inter-individually and intra-individually for all three ERTs.

### 2.4. Multiple Linear Mixed Model

**Figure 2:**
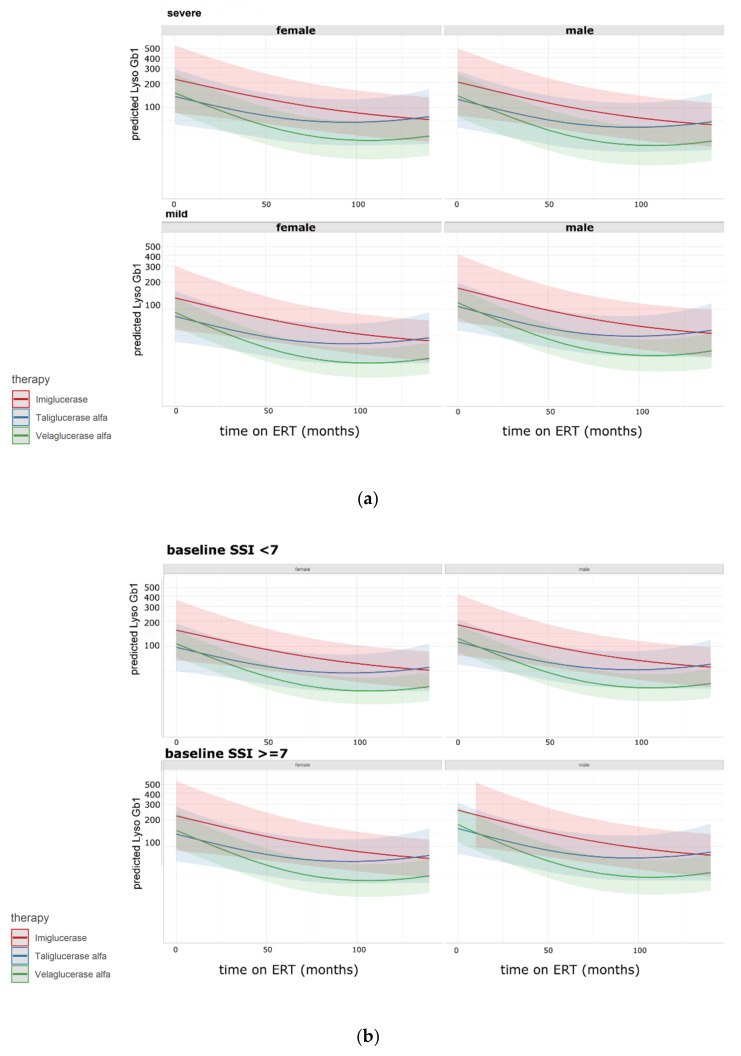
Model-based values for lyso-Gb1 by severity ((**a**) according to genotype, (**b**) according to severity score index (SSI)), enzyme replacement therapy (ERT) and sex. Model: a linear mixed model with a random intercept for individuals (135 individuals, 617 measures). Dependent variable: lyso-Gb1 log-transformed. Independent variables: age at the first visit, sex, ERT (Imiglucerase, Taliglucerase alfa, Velaglucerase alfa), SSI at baseline categorized (cut off 7), dosage, severity according to genotype, time on ERT. Time on ERT and type of ERT were entered as an interaction term and using natural cubic splines for the time on ERT with two degrees of freedom.

Figure 2 shows the marginal estimates and 95% CI of lyso-Gb1 by severity (according to genotype (a) and SSI (b)) and sex using a multiple linear mixed model. Figure 2b corresponds to the values reported in Table 3 and is based on the same regression model.

In all time points, lyso-Gb1 levels were higher for patients treated with imiglucerase than patients treated with velaglucerase alfa and taliglucerase alfa. However, a significant difference between velaglucerase alfa and imiglucerase was found only for the last three time points (Table 3). The fastest and steepest decrease in lyso-Gb1 levels was seen in patients treated with velaglucerase alfa (Figure 2). However, differences between ERTs were small.

## 3. Discussion

During the last decade, lyso-Gb1 has emerged as the most sensitive and specific biomarker for GD [12], outperforming other markers such as chitotriosidase or CCL18 and fulfilling almost all other characteristics of an ideal biomarker [13]. Lyso-Gb1 is relatively quick and easy to perform from DBS, reproducible, and inexpensive, its levels correlate with total disease burden and established clinical parameters, and it changes rapidly with the specific treatment [12,14]. Inter-individual and intra-individual variability of longitudinal lyso-Gb1 measurements may be related to the impact of the circadian rhythm, effects of nutrition and/or physical activity, or effects of coexisting pathological conditions [12,14], yet they are minimal and less dramatic compared to other biomarkers.

In fact, we have recently confirmed that its reduction following the initiation of ERT precedes the improvement of hematological parameters (platelet counts) and organ volumes (particularly decreased splenomegaly), and as such, it is capable of predicting response [15]. While the biomarker is expected to improve with therapy, it is important to demonstrate stability in untreated patients. We have recently reported a long-term follow-up (median 20 years) of 103 adult patients with GD1 who have never received ERT or SRT [16]. In this study, we showed a correlation between lyso-Gb1 and baseline disease severity. We used repeated measurements of lyso-Gb1 to confirm the patients’ stability and justify maintaining them with no specific treatment [16]. Similarly, in children with GD, lyso-Gb1 was shown to a reliable biomarker to monitor treated and untreated patients [6].

In the current study, we chose lyso-Gb1 measurements and compared, for the first time, the performance of the three enzymatic preparations currently commercially available in Israel (also in the USA, Canada, Australia, Latin and South American and more). The three ERTs are not biosimilar; similarities and dissimilarities between imiglucerase, velaglucerase alfa, and taliglucerase alfa have already been presented [8]. Specifically, velaglucerase alfa has a wild-type amino-acid sequence compared to imiglucerase and taliglucerase alfa, which have an amino acid substitution at position R496H. The substitution, although very mild [17], is still a disease-causing allele. Indeed, an in-vitro study comparing three human recombinant glucocerebrosidase enzymes (imiglucerase, wildtype and N370S mutated) has demonstrated a five-fold better specific activity of the wildtype and just three-fold increased specific activity for the imiglucerase compared to the N370S mutated one [18]. The internalization of the enzyme into macrophages is done via mannose receptor-mediated endocytosis. To achieve mannose termination, velaglucerase alfa is produced by culturing the fibrosarcoma cells in the presence of kifunensine, an α-mannosidase I inhibitor. This treatment results in the secretion of an enzyme whose N-glycosylation residues contain high mannosyl-type glycans, with six to nine (predominant) mannose residues each [19]. The at-least-twofold greater internalization rate of velaglucerase alfa compared to imiglucerase has been related to this high mannose type [20].

In a head-to-head randomized controlled study of naive GD patients, no patient receiving velaglucerase alfa developed antibodies, whereas four patients (23.5%) receiving imiglucerase developed IgG antibodies to imiglucerase, which were cross-reactive with velaglucerase alfa in one patient [21]. The rate of anti-drug antibody formation with taliglucerase was reported around 16–50% [22]. Differences in glycosylation patterns and manufacturing process from a human cell line [8] may explain the lower immunogenicity of velaglucerase alfa, contributing to its improved efficacy.

Heretofore no efficacy comparison beyond the first 12 months in the high-dose regimen [23] has been done. This comparison is of particular importance in Israel because of the use of the low-dose (15 units/Kg BW every-other-week) as a starting regimen for adult patients since the cost is a significant issue. Hence, if velaglucerase alfa (the wildtype sequence ERT) is also better in vivo than the other two (mutant ERTs), it will provide support for our practice to recommend a switch to velaglucerase alfa when there is a suboptimal response to any of the mutant ERTs rather than doubling their dose. Doubling the dose will be more costly and potentially associated with a higher risk of developing comorbidities such as metabolic syndrome [24].

Since July 2014, the Gaucher Unit at Shaare Zedek Medical Center has begun using lyso-Gb1 as the routine biomarker in all clinic visits. Until March 2020, a total of 3500 measurements (all anonymized) have been tested, of which 1017 were included in this study, i.e., samples of patients who have always been on the same ERT. Untreated patients, children, and those switching from one ERT to another or from ERT to SRT or the other way round were excluded to eliminate confounding factors.

The first part of the analysis was a comparison of baseline lyso-Gb1 levels between patients’ groups based on their demographic (sex and age), their treatment (ERT type, dose and duration), and their disease severity; the second set of statistical analyses was a comparison between the lyso-Gb1 values and overtime of a specific ERT product, adjusted for sex, disease severity and dosage. The association between the duration of therapy and lyso-Gb1 (higher levels among those patients treated for a shorter period) is self-explanatory, as the clearance of the storage glycolipids takes time (for example, in a post hoc analysis of the reduction in lyso-Gb1 in three clinical trials with velaglucerase-alfa, the mean percent reduction from baseline was 41% in 6 months, 64% at 12 months and 75% at 24 months [25]). Age at first time point observation was positively associated with lyso-Gb1 levels; in GD, as in many other lysosomal storage diseases and inherited metabolic disorders in general, the younger the age at presentation (the onset of symptoms), the more severe the phenotype is [1]. Additionally, younger patients had shorter times on ERT and, therefore, higher levels of lyso-Gb1.

A more interesting comparison, which is the primary rationale for the present study, is the lyso-Gb1 levels among the three ERTs. Those who believe that all ERTs are practically the same and even interchangeable may find support in the fact that the differences between the three enzymes were small. On the other hand, those who believe that velaglucerase alfa is somewhat better will appreciate the steeper and faster decreases of the lyso-Gb1 levels in velaglucerase alfa and the finding that the lyso-Gb1 levels at the last follow-up were lowest for velaglucerase alfa.

The limitations of the study are related to the retrospective nature of the data, the lack of randomization, possible biases associated with the selection of patients’ population, systematic differences in patient groups, and the low number of patients with pre-treatment (before initiation of ERT) measures of lyso-Gb1. Especially in patients treated with imiglucerase and taliglucerase alfa, only few data of the beginning of treatment were available (one patient treated with imiglucerase, two patients treated with taliglucerase alfa). In general, the different observation periods might yield imprecise estimates. While 135 subjects with a rare disease is a relatively high number, the uneven number of patients treated with each of the ERTs, the uneven number of patients with pre-treatment measurements, and the length of the follow-up may impact the results. Hence, more significant numbers and multicenter data will certainly lead to more powerful conclusions. As we do not foresee the chance of having a randomized study to answer this question, future studies will also need to rely on retrospective cohort data.

## 4. Materials and Methods

All 135 adult patients (age > 18 years) with GD1 seen at the Gaucher Unit, Shaare Zedek Medical Center, from July 2014 till March 2020 who were ever treated with a single ERT were included in the study, and their demographic and clinical characteristics are presented in Table 1. Patients who have been ever treated with more than one enzymatic preparation (“switched patients”) and those treated with substrate reduction therapy (SRT) were not included in the study. Three preparations of ERT are included in the study: imiglucerase (Cerezyme™, Sanofi/Genzyme, Cambridge, MA, USA), velaglucerase alfa (VPRIV™, Takeda, Glattpark-Opfikon (Zürich), Switzerland) and taliglucerase alfa (Elelyso™, Protalix/Pfizer, Carmiel, Israel and New York, NY, USA).

Genotyping was done by whole *GBA1* sequencing from DNA extracted from whole blood or dry blood spots (DBS) samples. The definitions of mild versus severe phenotype were determined by genotype (N370S (c.1226A>G) homozygous and N370S/R496H (c.1604G) compound heterozygous were categorized as “mild”, whereas all other genotypes as “severe”) and by the baseline severity score index (SSI) [26]. For the severity assessed by SSI, the patients were split into two groups, one group with SSI values lower than the median of 7 and one with SSI values higher or equal to the median.

Testing of lyso-Gb1 was performed on DBS samples collected during routine visits. Lyso-Gb1 levels were performed at Centogene™ according to the previously described method [11].

### Statistical Analysis

To report summary descriptive statistics, we used mean and standard deviation (SD) or median, interquartile range (IQR) for continuous variables, depending on the distribution. Additionally, we report the range of values as minimum and maximum. For nominal data, we report absolute and relative frequencies. Bivariate associations of different characteristics with lyso-Gb1 levels at first observation are reported as descriptive summary measures. Mann–Whitney U-Test and standardized mean difference (SMD) or Spearman rank correlation coefficient (ρ) were used for the association of two continuous variables. To assess differences in lyso-Gb1 over time and between treatments, linear mixed models with a random intercept for individuals were used. Lyso-Gb1 was log-transformed before the analysis to overcome the skewness of values. Models were adjusted for age at first observation, sex, SSI at the time of diagnosis (categorized, cut off 7), severity according to genotype (mild or severe), ERT dosage (categorized, cut off 15 unit/BW/dose), and time on ERT.

Additionally, an interaction term for time on ERT and type of ERT was included. To model the nonlinear relation between time on ERT and outcome, natural cubic splines were used for the time variable with two degrees of freedom. Marginal means of outcome values with 95% confidence intervals (CI) at different time points (time on ERT) and in different subgroups are reported and displayed graphically. Statistical analysis was done using SPSS version 26.0 [27] and R [28]. For statistical analyses, we used R packages ‘lme4’ [29], ‘r2glmm’ [30], ‘ggplot2’ [31], ‘ggeffects’ [32]. A two-sided significance level of α = 0.05 was considered. No adjustment for multiple testing was applied in this exploratory analysis. All p-values have therefore to be interpreted with caution. Interpretation of results is mainly based on effect sizes.

## 5. Conclusions

In conclusion, our report adds to the value of lyso-Gb1 as a reliable biomarker for monitoring treated patients with GD1, in this case, with ERT. The statistical models used herein may be adopted in future studies to help physicians, patients, and regulatory agencies comparing the various ERTs with the new therapeutic modalities, such as the existing SRTs (which are gaining popularity because of the oral route of administration [33]) or the investigational therapies, including pharmacological chaperones and gene therapy.

## Figures and Tables

**Table 1:** Characteristics of 135 patients with Gaucher disease type 1 who were treated with a single type of ERT.

	Total	Imiglucerase	Taliglucerase Alfa	Velaglucerase Alfa	p (SMD)
n	135	41	21	73	
Female, n (%)	77 (57%)	24 (58.5%)	11 (52.4%)	42 (57.5%)	0.891, (0.08)
Age, years ^1^: mean (SD), [range]	47 (17) [18–89]	48 (17) [18–84]	49 (17) [26–88]	46 (17) [18–89]	0.586, (0.13)
SSI: mean (SD), median (IQR), [range]	6 (4), 6 (4–8) [1–24]	8 (4) 8 (6–9) [1–19]	5 (2) 4 (3–5) [2–10]	6 (4) 5 (4–7) [2–24]	<0.001, (0.72)
Mild genotype ^2^, n (%)	88 (65.2%)	16 (39.0%)	18 (85.7%)	54 (74.0%)	<0.001, (0.72)
Dosage, ≤15 unit/kg/dose ^1^: n (%)	95 (70.4%)	34 (82.9%)	13 (61.9%)	48 (65.8%)	0.102, (0.32)
Time on ERT, months ^1^: median (IQR) [range]	51 (9–138) [0–322]	210 (139–246) [0–322]	51 (24–71) [0–118]	22 (2–58) [0–216]	<0.001, (1.40)
Observation time, months ^3^: mean (SD), [range]	32 (21) [0–66]	24 (21) [0–62]	35 (21) [0–64]	35 (19) [0–66]	0.020, (0.35)

SMD, standardized mean difference; SD, standard deviation; SSI, severity score index; IQR, interquartile range; ERT, enzyme replacement therapy. ^1^ At first observation time point. ^2^ Mild defined as c.1226A>G (N370S) homozygous or c.1604G (R496H)/c.1226A>G (N370S) heterozygous. ^3^ From first to last lyso-Gb1 measurement.

**Table 2:** Associations of covariables with lyso-Gb1 levels at the first time point observation.

Variable		n	Lyso-Gb1 (ng/mL) ^1^	*p* (SMD)
Sex	Males	58	79 (43–218)	0.3 (0.08)
	Females	77	67 (37–155)	
Age, years	≤35	39	92 (63–306)	0.03 (0.46)
	36–45	26	59 (34–94)	
	46–55	34	80 (26–183)	
	≥56	36	61 (36–181)	
Severity score index	<7	86	73 (36–171)	0.44 (0.15)
	≥7	49	77 (47–238)	
Genotype ^2^	Mild ^1^	88	71 (35–153)	0.127 (0.17)
	Severe	47	90 (53–253)	
Enzyme replacement therapy	Imiglucerase	41	72 (44–210)	0.55 (0.11)
	Taliglucerase	21	72 (27–131)	
	Velaglucerase	73	82 (42–193)	
Dosage	≤15 unit/kg/dose	95	72 (35–152)	0.24 (0.27)
	>15 unit/kg/dose	40	89 (46–222)	
Time on ERT, months	≤25	47	135 (65–251)	0.005 (0.41)
	26–55	22	70 (43–1105)	
	56–80	19	22 (36–126)	
	81+	47	66 (35–153)	

SMD, standardized mean difference; IQR, interquartile range. ^1^ median (IQR). ^2^ Mild; defined as c.1226A>G (N370S) homozygous. Severe; all other.

**Table 3:** Model ^1^ based lyso-Gb1 values by time on enzyme replacement therapy, sex, and severity score index.

Time on ERT	0-Month		12-Month		24-Month		48-Month		60-Month		72-Month	
N ^2^	40	7	54	10	43	7	72	19	40	9	38	9
SSI	SSI < 7	SSI ≥ 7	SSI < 7	SSI ≥ 7	SSI < 7	SSI ≥ 7	SSI < 7	SSI ≥ 7	SSI < 7	SSI ≥7	SSI < 7	SSI ≥ 7
Imiglucerase ^3^												
Female	174 (77–395)	247 (103–602)	153 (73–317)	217 (98–483)	133 (69–260)	191 (92–392)	104 (60–181)	148 (81–268)	93 (56–156)	132 (77–228)	84 (51–137)	119 (72–200)
Male	191 (83–437)	270 (112–659)	166 (78–347)	235 (106–518)	143 (74–279)	202 (99–416)	110 (63–191)	156 (86–281)	98 (58–164)	138 (80–240)	87 (53–144)	124 (74–209)
Taliglucerase ^3^												
Female	109 (57–206)	154 (77–305)	94 (54–166)	134 (72–247)	82 (49–137)	117 (67–206)	65 (41–104)	93 (55–156)	60 (37–95)	85 (50–144)	56 (35–90)	80 (47–136)
Male	118 (64–219)	169 (88–321)	101 (59–176)	145 (81–260)	88 (53–145)	125 (72–217)	69 (43–110)	98 (57–164)	62 (38–150)	88 (52–150)	58 (36–94)	82 (48–141)
Velaglucerase ^3^												
Female	120 (85–169)	171 (111–260)	95 (68–132)	134 (89–202)	75 (55–103)	107 (72–159)	50 (37–68)	71 (48–105)	43 (31–59)	60 (41–89)	37 (27–52)	54 (36–79)
Male	130 (90–191)	187 (120–287)	103 (72–145)	145 (96–219)	81 (57–113)	114 (76–171)	52 (38–74)	74 (50–111)	44 (32–62)	63 (43–94)	39 (28–55)	55 (37–82)
*p* values ^4^	Imi vs. Tali: 0.592 Imi vs. Vela: 0.635 Tali vs. Vela: 0.943		Imi vs. Tali: 0.502 Imi vs. Vela: 0.399 Tali vs. Vela: 1.000		Imi vs. Tali: 0.413 Imi vs. Vela: 0.190 Tali vs. Vela: 0.922		Imi vs. Tali: 0.307 Imi vs. Vela: 0.020 Tali vs. Vela: 0.460		Imi vs. Tali: 0.311 Imi vs. Vela: 0.006 Tali vs. Vela: 0.291		Imi vs. Tali: 0.356 Imi vs. Vela: 0.003 Tali vs. Vela: 0.189	

ERT, enzyme replacement therapy; SSI, severity score index; Imi, Imiglucerase; Tali, Taliglucerase alfa; Vela, Velaglucerase alfa. ^1^ Model: a linear mixed model with a random intercept for individuals (135 individuals, 617 measures). Dependent variable: lyso gb1 log-transformed, independent variables: age at the first observation time-point, sex, ERT (Imiglucerase, Taliglucerase, Velaglucerase), SSI at baseline categorized (cut off 7), dosage, severity according to genotype, time on ERT; and therapy was entered as interaction term and with using natural cubic splines for the time on ERT with two degrees of freedom. ^2^ For calculating the number of measurements, the following periods were used: 0: 0–3 month, 12: 4–12 month, 24: 13–24 month, 48: 25–48 month, 60: 49–60 month, 72: 61–72 month, additionally 269 measures of 73+ months were used in the model. ^3^ model-based marginal means and 95%CI (back-transformed form log-transformed values). ^4^ adjusted within each time point according to Tukey’s method.

## Data Availability

Data cannot be shared due to ethical and privacy issues.
